# Family care reduces the incidence of neonatal sepsis: A systematic review and meta-analysis

**DOI:** 10.3389/fped.2023.1089229

**Published:** 2023-04-12

**Authors:** Niloofar Ganji, Mashriq Alganabi, Masaya Yamoto, Sinobol Chusilp, Agostino Pierro, Bo Li

**Affiliations:** Division of General and Thoracic Surgery, Translational Medicine Program, The Hospital for Sick Children, Toronto, ON, Canada

**Keywords:** neonatal sepsis, family care, kangaroo care, sepsis, necrotizing entercolitis

## Abstract

**Purpose:**

Family-involved care in the neonatal intensive care unit (NICU) helps to alleviate neonatal anxiety and promotes breastmilk intake, body growth and neurological development, but its effect on reducing the incidence of neonatal sepsis is not known. We conducted a systematic review and meta-analysis of randomized controlled trials (RCT) to evaluate whether neonates receiving family care have a lower incidence of neonatal sepsis compared to neonates receiving standard NICU care.

**Methods:**

MEDLINE, Embase, Web of Science, and CENTRAL were searched for RCTs that compared preterm neonates receiving family care vs. standard NICU care. From 126 articles that were identified and screened, 34 full-text articles were assessed for eligibility, and 5 RCTs were included. The primary outcome was the development of sepsis. The RevMan 5.4 software was used to conduct the Meta-analysis.

**Results:**

The metanalysis, based on 5 RCTs demonstrated that neonates receiving family-involved care had significantly lower incidence of sepsis (12.0% vs. 16.3%), increased body weight, and reduced length of hospital stay compared to those receiving standard NICU care.

**Conclusion:**

This study suggests that family-involved care in NICU can (i) reduce the incidence of neonatal sepsis, (ii) improve growth, and (iii) reduce the length of hospital stay. This study highlights the need for evaluating whether family-involved care improves other neonatal outcomes.

## Introduction

Neonatal sepsis is a condition characterized by systemic bacterial, viral, or fungal (yeast) inflammation, accompanied by hemodynamic changes and can result in high morbidity and mortality. A recent systematic review calculated that in 12 countries between 1979 and 2016, the incidence of sepsis in neonates was 2202 per 1 00 000 live births, with mortality between 11% and 19% (1). The most important neonatal risk factor for sepsis are prematurity or low birth weight. The incidence of infection is 3–10× greater in preterm low birth weight (LBW) infants compared to full-term and normal birthweight infants (2–4). Impaired intestinal epithelial barrier function and immune dysfunction in preterm infants further increase the risk of infection (4). Additionally, preterm infants often need intravenous access for prolonged times, endotracheal intubation, or other procedures which provide a portal of entry and therefore influence the physical barrier and clearance mechanisms; as a result, preterm infants are at an increased risk for infections (3).

The risk of neonatal mortality and morbidity is higher in infants born before term or at low birth weight (LBW) including hindered growth and development as well as chronic diseases (5, 6). Health technologies such as incubators can help improve outcomes in high-risk infants. However, depriving neonates from their parents (maternal separation) has recently been shown to contribute to the development of various neonatal intestinal disorders (7). Maternal separation affects intestinal epithelial barrier integrity which is vital in protecting against infection and is one of the key events in initiating pathological processes in the intestine. Preclinical studies have shown that single or repeated episodes of maternal separation in the early life of rodents leads to increased colonic and ileal permeability, barrier dysfunction, and increased bacterial adherence to the mucosal layer (3, 8–10). However, whether the incidence of neonatal sepsis is altered by maternal separation is not yet known.

The family integrated care in the neonatal intensive care unit (NICU) has been established to ensure that parents can be maximally involved in the care of their infants and provide the physiological and emotional support that their vulnerable neonates need. This program has been implemented in various countries and been achieved by using various skin-to-skin contact (SSC), kangaroo mother care (KMC) and/or parents caregiving. KMC and increased SSC help maintain the infant's temperature and other vital signs stable, especially through breastfeeding (11). These are important benefits for all newborns and especially for those who are preterm (12).

There may be additional benefits to KMC or other forms of increased skin-to-skin contact. A study in the UK demonstrated that an actual intervention to increase breastfeeding, KMC and SSC in neonatal units was cost-effectiveness and clinically advantageous (13). Additionally, a literature review examined the effects of kangaroo care on infant weight gain (14). Patients examined in this study were low-birth weight preterm infants without any respiratory distress, infections, invasive respiratory support, or major anomalies. The study found that Kangaroo care significantly increased weight gain secondary to increased breast milk intake.

Whether family-involved care in the NICU affects the incidence of neonatal sepsis is not known. Based on the evidence illustrated above, we sought to examine whether increased SSC between parents and infant reduces the incidence of neonatal sepsis. To evaluate this, we conducted a systematic review and meta-analysis on currently available data.

## Method

### Search strategy

PubMed, Embase, Web of Science, and the Cochrane Library (CENTRAL) databases were searched using for the search terms as shown in Table 1 up to July 3, 2021. Language restrictions were not implemented when searching for articles. The Hospital for Sick Children library resources were used to obtain search results.

**Table 1:** Search terms used for pubMed, embase, Web of science, and the cochrane library (CENTRAL) databases.

**PubMed**
• newborn* or newly born* or neonat* or prematur* or preterm* or pre-term* AND • “skin to skin” OR “chest to chest” OR kangaroo OR “maternal care” OR “mother care” OR “paternal care” OR “parental care” OR “family care” OR “maternal involvement” OR “parental involvement” OR “family involvement” OR “mother integrated” OR “family integrated” OR “care by parent” OR “family center” OR “family centered care” AND • neonatal sepsis or sepsis
**Embase**
• newborn* or newly born* or neonat* or prematur* or preterm* or pre-term* AND • “skin to skin” OR “chest to chest” OR kangaroo OR “maternal care” OR “mother care” OR “paternal care” OR “parental care” OR “family care” OR “maternal involvement” OR “parental involvement” OR “family involvement” OR “mother integrated” OR “family integrated” OR “care by parent” OR “family center” OR “family centered care” AND • neonatal sepsis or sepsis
**Web of Science**
• newborn* or newly born* or neonat* or prematur* or preterm* or pre-term* AND • “skin to skin” OR “chest to chest” OR kangaroo OR “maternal care” OR “mother care” OR “paternal care” OR “parental care” OR “family care” OR “maternal involvement” OR “parental involvement” OR “family involvement” OR “mother integrated” OR “family integrated” OR “care by parent” OR “family center” OR “family centered care” AND • neonatal sepsis or sepsis
**Cochrane Library (CENTRAL)**
• newborn* or newly born* or neonat* or prematur* or preterm* or pre-term* AND • “skin to skin” OR “chest to chest” OR kangaroo OR “maternal care” OR “mother care” OR “paternal care” OR “parental care” OR “family care” OR “maternal involvement” OR “parental involvement” OR “family involvement” OR “mother integrated” OR “family integrated” OR “care by parent” OR “family center” OR “family centered care” AND • neonatal sepsis or sepsis

### Study selection criteria

The inclusion and exclusion criteria were determined and established before the review. Inclusion criteria were preterm infants, direct comparison of family involved care (such as SCC, KMC) vs. standard NICU care, care provided in the NICU, patients recruited to both arms of the studies being at the same time, in all languages. Exclusion criteria were no direct comparison between family involved care and standard NICU care, neonatal sepsis occurring before the intervention, and no full text being available.

### Study selection process

The search results from PubMed, Embase, Web of Science, and the Cochrane Library (CENTRAL) database were combined, duplicate articles were removed. Papers were then screened for eligibility based on the inclusion and exclusion criteria. For screening, titles and abstracts were used by two independent authors (BL and MY). The same two independent authors reviewed full texts and selected the papers to be included. All disagreements were discussed and resolved by consensus agreement.

### Outcome assessment

The incidence of neonatal sepsis was the primary outcome and was directly compared between NICU patients receiving family involved care and those receiving standard NICU care. Secondary outcomes included length of hospital stay, weight gain, and incidence of necrotizing enterocolitis (NEC).

### Data extraction

Included articles underwent full text review and data extraction. Two independent authors (BL and MY) evaluated each of the papers, and extracted data: author, study design, location/country of the study, age of the patients, data and results on the incidence of neonatal sepsis as primary outcome, and secondary outcome of length of hospital stay, weight gain, and NEC incidence. The authors held follow-up meetings to validate the data.

### Quality assessment

The Risk of Bias 2 (RoB2) tool for assessing risk of bias in randomized control trials was used (15) in this review. This is the recommended tool to assess the risk of bias in randomized control trials included in Cochrane Reviews. It contains a fixed set of domains of bias, focusing on different aspects of trial design, conduct and reporting. In each domain, judgement is made on the level of risk and categorized into “low risk”, “some concern”, or “high risk”.

### Statistical analysis

Pooled odds ratios (OD) and their respective 95% confidence intervals (CI) were utilized for dichotomous variables. Weighted Mean Difference (WMD) and their 95% CI were utilized for continuous variables using inverse variance. Heterogeneity of data was assessed using *I*^2^. A fixed effect model was used if *I*^2^ < 50% and a random effect model was used if *I*^2^ ≥ 50%. Statistical analysis was conducted using Review Manager 5.4 (Cochrane Collaboration). A *P*-value of ≤0.05 was considered significant.

## Results

### Literature search

**Figure 1:**
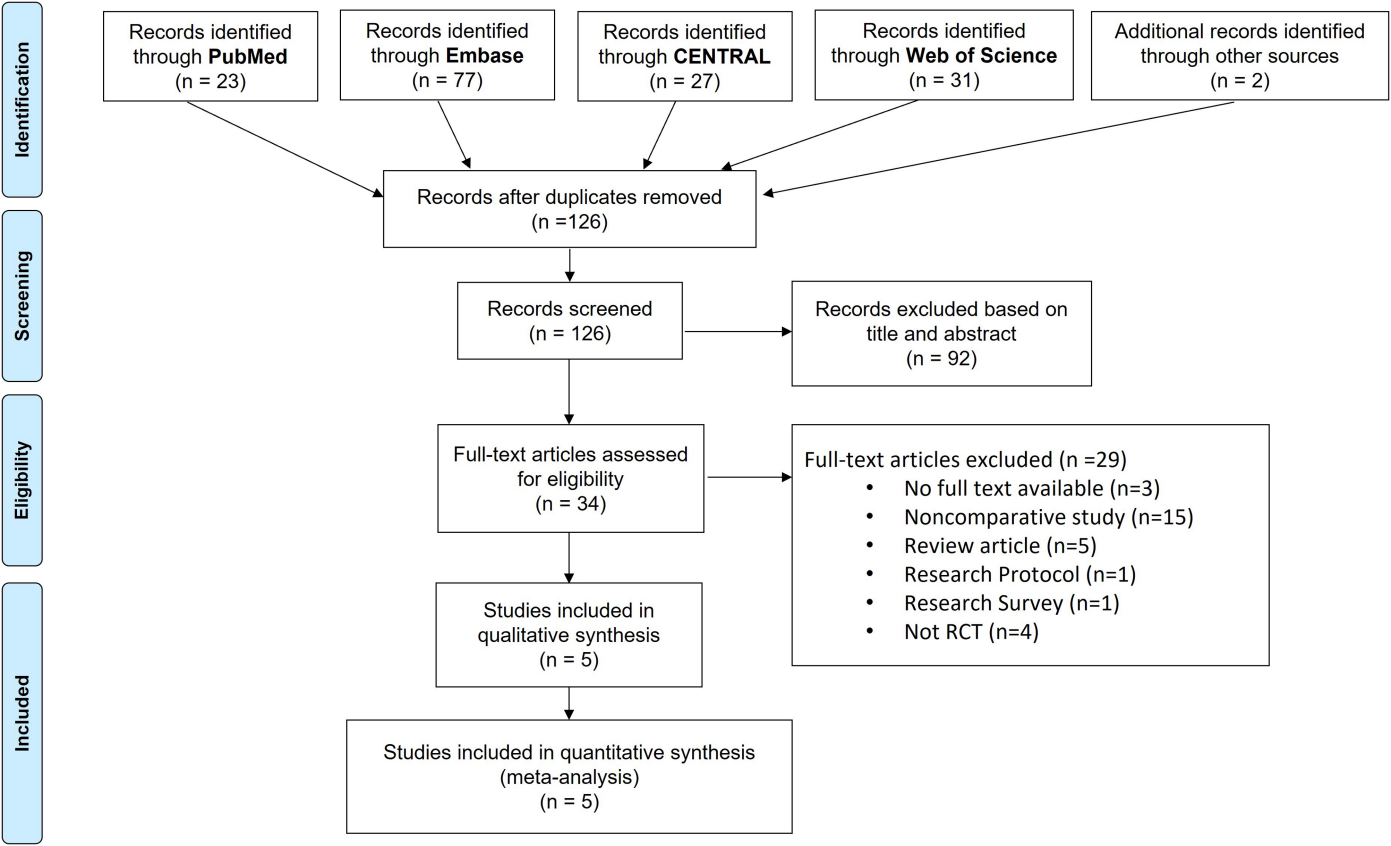
PRISMA flowchart of article selection for the systematic review. Five RCTs that directly compared NICU patients receiving standard NICU care to additional family care was identified and included in the meta-analysis.

PRISMA guidelines were followed for the study search and selection (16). From the PubMed, Embase, Web of Science, and the Cochrane Library (CENTRAL) databases, 124 records were yielded after removal of duplicates. After title and abstract screening, 92 articles were excluded. 29 additional articles were excluded during the full text review due to one or more of the following reasons: no full text available (*n* = 3), non-comparative study (*n* = 15), review article (*n* = 5), research protocol (*n* = 1), research survey (*n* = 1) or not RCT (*n* = 4). Five RCT studies (17–21) were included in the qualitative and quantitative analyses (Figure 1).

### Study and patient characteristics

All studies included were RCTs with preterm infants cared in the NICU. There were infants from 3 countries (China Taiwan, Sweden and USA) that were included in the analyzed study summarized in Table 2. All studies directly compared infants in the family integrated NICU care vs. standard NICU care were examined.

**Table 2:** Included RCTs and their study characteristics.

Author	Country	Population	Intervention		Primary outcome measurement
			Family Care	Standard Care	
Chen et al 2013	Taiwan	birth weight <1,500 g; gestational age <37 weeks;	Family-Centered Care program	Usual care program	Morbidity, growth and neurodevelopmental parameters
Ortenstand et al 2009	Sweden	preterm birth (37 weeks), no major congenital malformation diagnosed on admission	Family care (FC)	Standard NICU care	Total length of hospital stays
Rojas et al 2003	USA	Less than 32 weeks of gestation, less than 1,500 g, minimal ventilatory support, and hemodynamically stable	Skin-to-Skin Care Group	Traditional Holding Group	Weight, crown heel Length, head circumference
Welch et al 2013	USA	infants of 26–34 weeks gestation	Family Nurture Intervention in the NICU	Standard NICU care	Assessments of length of stay, feasibility, and safety
Yu et al 2017	Taiwan	a birth weight <1,500 grams and gestational age <37 weeks without congenital abnormalities or severe perinatal and neonatal diseases	Family-centered intervention program	Usual care program	Primary outcome of neurodevelopment

### Primary outcome

**Figure 2:**
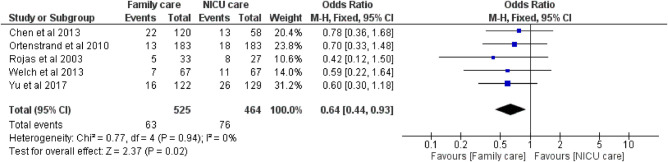
Neonatal sepsis incidence. Incidents of neonatal sepsis in the standard NICU care group vs. the additional family care group were compared. There was a significantly decreased incidence of neonatal sepsis in the family care group with an odds ratio of 0.64 [95% CI: 0.44, 0.93], *P* = 0.02.

Incidence of neonatal sepsis was extracted as the primary outcome measure from the included studies. There was significantly higher incidence of neonatal sepsis in the NICU care patients than those that received additional family care. Among the RCT studies 76/464 (16.3%) of the patients receiving NICU care developed sepsis compared to 63/525 (12.0%) among patients receiving family care (Figure 2).

### Secondary outcomes

**Figure 3:**
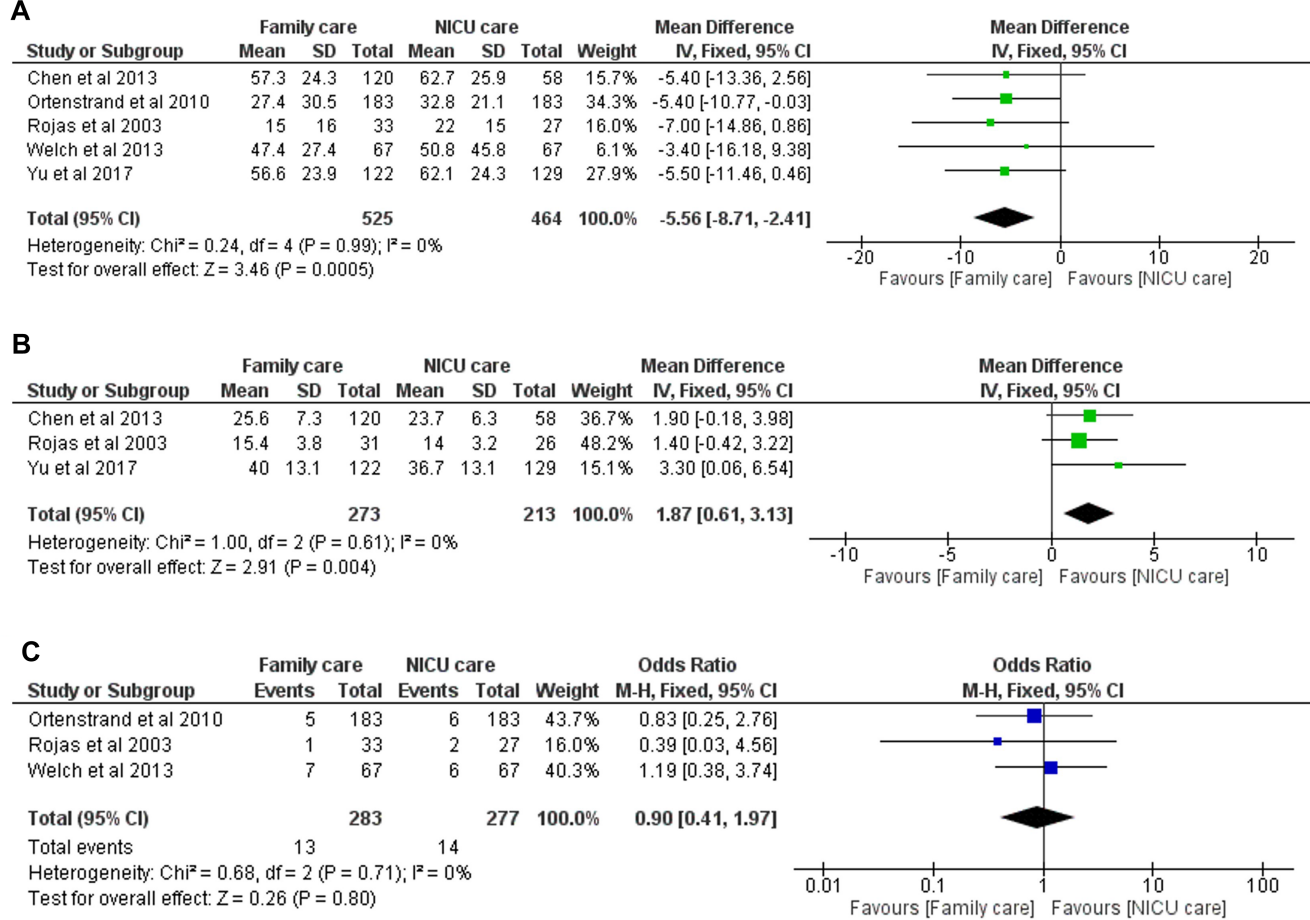
Assessment of secondary outcomes. **A**. Length of hospital stay was significantly shorter in the family care group than the NICU care group −5.56 days [95%CI: −8.71 days, −2.42 days], *P* = 0.0005. **B**. Body weight gain was significantly greater in the family care group compared to the NICU group 1.87 grams per day [95%CI: 0.61, 3.13], *P* = 0.004. **C**. There was no significant difference in the incidence of necrotizing enterocolitis (NEC) between the NICU care group and the family care group, *P* = 0.8.

Three secondary outcomes including length of hospital stay, body weight, and incidence of NEC, were extracted from the included studies. There was a significantly longer length of hospital stay in the NICU care group compared to the family care group MD −5.56 days [95%CI: −8.71, −2.41] (Figure 3A). Body weight gain was significantly lower in the NICU care group compared to the family care group, MD 1.87 grams per day [95%CI: 0.61, 3.13] (Figure 3B). There was no significant difference in the incidence of NEC between the NICU care group and the family care group (Figure 3C).

### Risk of bias assessment

**Figure 4:**
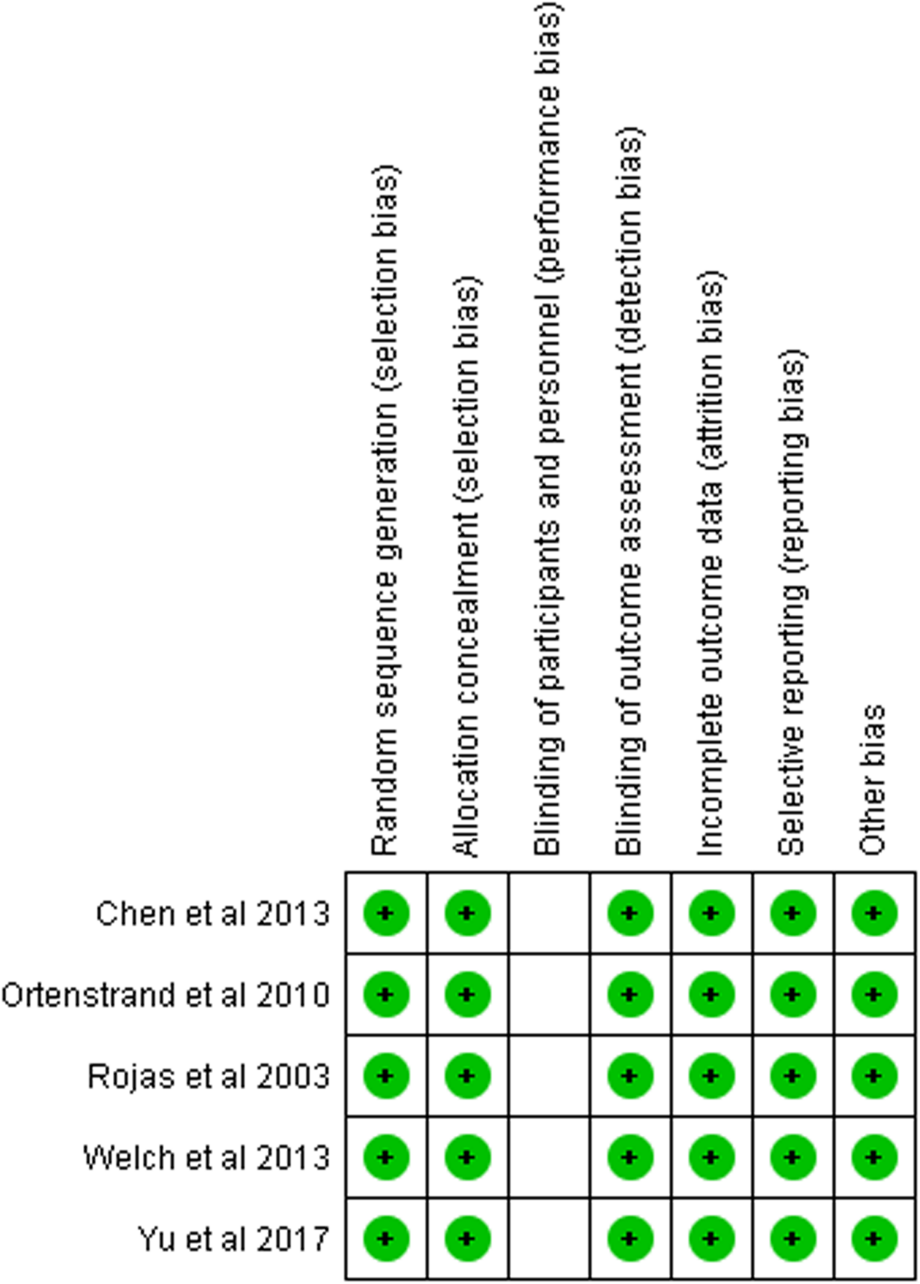
Risk of bias (RoB2) assessment of the included RCTs. All the studies had appropriate randomization, allocation, blinding of outcome assessment, no attrition, and included all patients within specific time windows of the respective studies eliminating potential selection bias. However, all the included studies did not have blinding of the care received to either the care providers or the parents as it is not possible to do. The overall risk of bias among all the included studies was low.

RoB2 was used to assess the risk of bias associated with the included RCT studies (Figure 4). The included studies had a low level of risk in all examined parameters except for blinding of the participants and healthcare personnel. It is not possible to blind the parents, or the healthcare personnel directly involved as the intervention is involving the family in the care of the child. The overall risk of bias among all the included RCTs is low.

## Discussion

In this study, five RCTs were included with a total of 989 neonatal NICU patients. There was a significantly higher incidence of neonatal sepsis among patients receiving standard NICU care in comparison to those receiving additional family care (Figure 2). Patients receiving family care also had shorter length of stay at the hospital and developed larger body weight gain (Figure 3). The quality of the evidence was high (Figure 4) as all included studies were RCTs that had appropriate randomization and patient follow-up.

It is common practice to nurse preterm neonates separate from their caregivers and in incubators where they are provided with oxygenation and their vital signs are consistently monitored. There are multiple potential factors involved in maternal separation which induce neonatal intestinal injury including impairment of intestinal epithelial barrier function, alterations in microbiome, oxidative stress, endoplasmic reticulum stress, and gut inflammation (7). The exact mechanisms by which maternal separation alters normal physiological processes remains to be elucidated. Nevertheless, defects in intestinal barrier function and gut inflammation caused by maternal separation may increase the susceptibility of the preterm intestine to developing neonatal sepsis.

More and more NICUs are building single family rooms to accommodate parents to be present continuously during the day and at night with their infant. There is a growing body of evidence that supports family involved NICU care in LBW infants as a beneficial alternative to conventional neonatal care. A systematic review found that compared to conventional care, family care reduced severe infection, nosocomial infection/sepsis, lower respiratory tract disease, hypothermia, severe illness, and mortality at discharge as well as increased body weight, body length, and head circumference gain (22). Additionally, a systematic review and meta-analysis including randomised and non-randomised studies by Veenendaal et al. showed lower incidences of sepsis and increased exclusive breastfeeding rates upon discharge and no difference in long-term neurodevelopment for preterm infants hospitalized in family room care compared with traditional NICU (23). Consistently, investigating clinical outcomes of preterm infants receiving family-care in the NICU vs. those receiving standard NICU care, we found reduced sepsis incidence and increased weight gain in preterm neonates.

As a secondary outcome, we also compared the incidence of NEC between the family care vs. standard NICU care groups but did not find a significant difference. Preclinical studies have shown that early maternal separation leads to gut injury resembling NEC (9, 24–26). Furthermore, as demonstrated by clinical studies, there was a lower incidence of NEC when parents provided most of their infant's care in the NICU (27, 28). Interestingly, although Welsh et al. did not find a difference in NEC incidence between standard NICU care and family care, NEC progression towards more advanced NEC requiring surgical intervention occurred in 2 of the 6 confirmed NEC neonates receiving standard NICU care and in none of the 7 NEC confirmed neonates who received family care (18). A major benefit of family care is increasing breast milk intake (12). Preterm infants that receive human breastmilk have a lower incidence of NEC compared to those fed preterm formula (29). This can be due to breastmilk containing possible bioactive substances with immune-modulating and/or bactericidal activity (30). We have previously shown that administration of human breast milk-derived exosomes during experimental NEC promoted growth of intestinal epithelial cells and attenuated damage in the intestine (31, 32). Additionally, we have also shown that human milk oligosaccharides derived from human breast milk increase mucin expression (33) and activate intestinal cell differentiation (34), which together protect against experimental NEC. Hence, future prospective RCTs are needed to further investigate the effect of family-involved care on the incidence of NEC.

Taken together, this review suggests that the addition of family care or skin-to-skin care reduced the incidence of neonatal sepsis, improved the outcome of the NICU patients, and shortened the duration of their hospital stay. Future prospective RCTs are needed to investigate the effect of family-involved care on other neonatal outcomes as well as on the incidence of neonatal diseases such as NEC.

## Data Availability

The original contributions presented in the study are included in the article/Supplementary Material, further inquiries can be directed to the corresponding author.
